# Dietary iron intake predicts all-cause and cardiovascular mortality in patients with diabetes

**DOI:** 10.1038/s41387-024-00286-2

**Published:** 2024-08-23

**Authors:** Chenchen Yang, Tingting Hu, Chenglin Li, Aifeng Gong

**Affiliations:** 1 Department Emergency, The Affiliated Huai’an No.1 People’s Hospital of Nanjing Medical University, Huai’an, 223300 China; 2 Department Cardiology, The Affiliated Huai’an No.1 People’s Hospital of Nanjing Medical University, Huai’an, 223300 China; 3 Department Cardiothoracic Surgery, The Affiliated Huai’an No.1 People’s Hospital of Nanjing Medical University, Huai’an, 223300 China; 4 Department General Practice, The Affiliated Huai’an No.1 People’s Hospital of Nanjing Medical University, Huai’an, 223300 China

**Keywords:** Endocrinology, Cardiovascular diseases

## Abstract

**Background:**

Limited data exists on the link between dietary iron intake and mortality in diabetes. Our investigation aimed to explore how dietary iron intake correlates with overall and cause-specific mortality in diabetic individuals.

**Methods:**

This analysis encompassed 5970 participants with diabetes from the National Health and Nutrition Examination Survey spanning 1999 to 2014. Baseline data were collected through surveys and examinations, with mortality status tracked via National Death Index records until December 31, 2015. Cox proportional hazard models were utilized to calculate hazard ratios (HR) and 95% confidence intervals (CI) for mortality from various causes, including cardiovascular disease (CVD) and cancer.

**Results:**

The average iron intake among the cohort was 14.1 ± 7.4 mg daily, with an average participant age of 61.3 and 3059 (51.3%) male adults. Over 41,425 person-years of follow-up, 1497 deaths were recorded. Following adjustments for multiple variables, an iron intake between 11.1 and 14.4 mg was associated with the lowest risk of all-cause mortality (HR 0.83 [0.70, 0.99], *P* < 0.05) compared to the reference group (<8.3 mg). Analysis of dose-response curves revealed an L-shaped pattern in men and a J-shaped pattern in women concerning the relationship between iron intake and all-cause mortality.

**Conclusions:**

Our findings suggest a nonlinear association between dietary iron intake and all-cause mortality in individuals with diabetes. Specifically, higher iron intake may increase all-cause mortality risk in men, while potentially exert a protective effect in women.

## Introduction

Diabetes mellitus is a prevalent global health concern, with approximately 451 million individuals affected in 2017. Projections from the International Diabetes Federation indicate that this number is expected to rise significantly, reaching an estimated 693 million individuals by 2045 [1]. Diabetes has been linked to a heightened risk of both all-cause mortality and cardiovascular mortality [2]. It is of great significance to identify factors to delay the progress of diabetes and reduce mortality.

Iron serves as a vital component for cellular energy metabolism, oxygen transportation, and numerous enzymatic processes crucial for life. Imbalances in iron levels, whether deficiency or excess, are associated with the development of various diseases [3–5]. Several studies have indicated a potential link between increased iron intake and a higher risk of diabetes across diverse populations. For instance, a cross-sectional survey conducted in China revealed that elevated heme iron intake, rather than total or non-heme iron intake, was associated with an increased risk of diabetes. However, establishing a causal relationship warrants further investigation [6]. In a prospective cohort study involving 38,394 participants, findings supported a positive correlation between heme iron intake derived from red meat consumption and the risk of developing type 2 diabetes [7]. Non-heme iron was inversely associated with incidence of type 2 diabetes while heme iron displayed the positive relationship in postmenopausal women [8]. Besides, iron content also influenced the risk of diabetic complications [9], such as diabetic retinopathy [10], kidney injury [11] and diabetic peripheral neuropathy [12]. Nevertheless, scarce studies elucidated the relationship between iron and mortality in diabetes.

In this study, we aimed to investigate the relationship between dietary iron intake and all-cause or cause-specific mortality in diabetic individuals.

## Methods and materials

### Study population

The research utilized data from the National Health and Nutrition Examination Survey (NHANES) spanning the years 1999 to 2014, which is a comprehensive nationwide survey aimed at evaluating the health and nutritional status of both adults and children across the United States. Participants diagnosed with diabetes were identified based on self-reported medical history, usage of hypoglycemic medications, or specific biochemical criteria, including serum HbA1c levels exceeding 6.5% or fasting serum glucose levels surpassing 7.0 mmol/L, as outlined below. Following the exclusion of individuals below 18 years of age (*n* = 109) and those lacking records of iron intake (*n* = 26), a total of 5970 diabetic participants were included in the analysis. Approval for the study was obtained from the institutional review board of the National Center for Health Statistics (Protocol #98-12), and written informed consent was obtained from all participants prior to their involvement in the study.

### Assessment of dietary intake

Detailed dietary intake information, encompassing the types and quantities of foods and beverages consumed, was gathered through a 24-h dietary recall method. To ensure accuracy, a multi-pass approach was employed to meticulously document iron intake. This method involved multiple passes of questioning to clarify and validate the reported intake of iron-rich foods and beverages. (https://www.cdc.gov/nchs/nhanes/measuring_guides_dri/measuringguides.htm). Trained interviewers conducted the interviews prior to the physical examination, utilizing the Computer-Assisted Personal Interview system, within the participants’ homes. During this process, detailed dietary information was collected. Subsequently, trained nutritionists meticulously reviewed the gathered data, cross-referencing reported dietary supplement entries with known supplements listed in the in-house NCHS Product Label Database to ensure accuracy and completeness (https://wwwn.cdc.gov/Nchs/Nhanes/2013-2014/DS1TOT_H.htm). The total energy and iron intake were calculated according to the dietary intake.

### Assessment of outcome

The primary outcome of interest in the study was all-cause mortality, while secondary outcomes included mortality specifically attributed to cardiovascular disease and malignant neoplasms. Mortality status was determined through linkage to the National Death Index, with data collected up to December 31, 2015. Cardiovascular disease-related mortality was defined using International Classification of Diseases, Tenth Revision (ICD-10) codes I00-I09, I11, I13, or I20-I51, while malignant neoplasm-related mortality was defined by ICD-10 codes C00-C97.

### Assessment of variates

Baseline characteristics of participants were obtained through a combination of questionnaires and physical examinations, encompassing various aspects such as demographics (including sex, age, race, educational level, family poverty income ratio), lifestyle factors (such as physical activity, alcohol consumption, smoking), medical history (including hypertension and cardiovascular diseases), and medication usage (such as antihypertensive drugs, hypoglycemic drugs, and lipid-lowering drugs). Biomedical measurements were conducted as follows: Plasma fasting glucose levels were measured using an enzymatic method, while HbA1c levels were determined through an HPLC method. Triglycerides and LDL cholesterol levels were enzymatically assessed using the Roche Modular P chemistry analyzer. Estimated glomerular filtration rate (eGFR) was calculated using the Chronic Kidney Disease-Epidemiology Collaboration (CKD-EPI) equation. Race was categorized as non-Hispanic white, non-Hispanic black, Mexican American, other Hispanic, or others. Educational attainment was grouped into less than high school, high school or equivalent, and college or above. Poverty income ratio (PIR) was calculated as the ratio of household income reported by the participant to the appropriate poverty threshold for household size, categorized as <1, 1–3, and >3. Smoking status was classified as current, past, or never. Physical activity status was categorized as vigorous, moderate, or inactive. Hypertension was defined as a history of hypertension, blood pressure readings ≥140/90 mm Hg, or the use of antihypertensive medications. Cardiovascular disease (CVD) was defined based on self-reported conditions, including congestive heart failure, coronary heart disease, angina pectoris, heart attack, and stroke. For variables with missing values in Cox proportional hazards regression models, imputation was performed using the predictive mean matching method, where the observation with the closest predicted transformed value served as the donor.

### Statistical analysis

Continuous variables were expressed as mean ± standard deviation, while categorical variables were presented as counts and proportions (percentage). Iron intake was divided into five groups based on quintiles, and differences between these groups were assessed using one-way analysis of variance (ANOVA) for continuous variables or chi-square tests for categorical variables. Univariate survival analysis was conducted using Kaplan–Meier analysis and the Log-rank test. Multivariate Cox proportional hazards regression models were utilized to estimate hazard ratios (HRs) and 95% confidence intervals (CIs) for all-cause, cardiovascular, and cancer mortality. Three models were employed for adjustment: Model 1 included age and gender; Model 2 further adjusted for race, education level, poverty income ratio (PIR), body mass index (BMI), smoking status, alcohol consumption, and physical activity; Model 3 additionally adjusted for hypertension, cardiovascular disease (CVD), estimated glomerular filtration rate (eGFR), baseline iron levels, and serum iron levels. The nonlinear relationship between iron intake and all-cause mortality was described using restricted cubic splines. Subgroup analysis was conducted to investigate the effect of baseline serum iron levels on this relationship. All statistical analyses were performed using R version 3.6, with statistical significance set at *P* < 0.05.

## Results

The current study comprised 5970 participants diagnosed with diabetes. Within this cohort, the average daily iron intake stood at 14.1 ± 7.4 mg, with an average age of 61.3 years. Among the participants, 3059 (51.3%) were male adults. Notably, individuals with elevated iron intake tended to be male and non-Hispanic, with a higher poverty income ratio (PIR), serum iron levels, and energy intake compared to those with lower iron intake levels (Table 1).

**Table 1:** The characteristics of diabetic patients according to the iron intake quintiles.

Variable	F1 (1194)	F2 (1195)	F3 (1194)	F4 (1194)	F5 (1193)	*P*
Iron intake range	<8.3	8.3–11.1	11.1–14.4	14.4–18.8	>18.8	-
Iron intake, mg	6.3 (1.6)	9.7 (0.8)	12.7 (0.9)	16.4 (1.3)	25.6 (7.1)	<0.001
Male, %	442 (37.1)	505 (42.3)	610 (51.1)	673 (56.4)	829 (69.5)	<0.001
Age, years	62.7 (13.5)	62.6 (13.7)	60.9 (13.9)	60.4 (14.0)	60.0 (14.2)	<0.001
Race, %						<0.001
Non-Hispanic white	353 (29.6)	423 (35.4)	448 (37.5)	489 (41.0)	554 (46.4)	
Non-Hispanic black	404 (33.9)	332 (27.8)	320 (26.8)	300 (25.1)	245 (20.5)	
Mexican American	251 (21.1)	261 (21.8)	247 (20.7)	243 (20.4)	241 (20.2)	
Others	184 (15.4)	179 (15.0)	179 (15.0)	162 (13.6)	153 (12.8)	
Education, %						<0.001
Less than high school	634 (53.6)	506 (42.8)	459 (38.7)	401 (33.8)	380 (32.1)	
High school or equivalent	247 (20.9)	259 (21.9)	285 (24.1)	269 (22.7)	292 (24.6)	
College or above	302 (25.5)	417 (35.3)	441 (37.2)	516 (43.5)	513 (43.3)	
PIR, %						<0.001
<1	350 (33.4)	281 (25.4)	232 (21.3)	218 (20.0)	221 (20.1)	
1–3	492 (47.0)	548 (49.5)	533 (48.9)	501 (46.0)	491 (44.6)	
>3	205 (19.6)	278 (25.1)	325 (29.8)	371 (34.0)	388 (35.3)	
BMI, kg/m2	32.0 (7.16)	32.0 (7.06)	32.2 (7.44)	32.5 (7.74)	32.0 (7.46)	0.478
Drinking, %	246 (47.6)	241 (51.4)	216 (52.4)	223 (55.2)	179 (50.7)	0.233
Smoking, %						0.109
Current	188 (23.0)	166 (21.4)	182 (23.8)	150 (19.2)	168 (23.3)	
Past	31 (3.8)	23 (3.0)	33 (4.3)	28 (3.6)	38 (5.3)	
Never	600 (73.3)	586 (75.6)	550 (71.9)	604 (77.2)	515 (71.4)	
Activity, %						<0.001
Inactive	121 (27.2)	99 (19.5)	123 (23.4)	113 (18.8)	87 (15.5)	
Moderate	207 (46.5)	271 (53.3)	263 (50.1)	307 (51.1)	285 (50.8)	
Vigorous	117 (26.3)	138 (27.2)	139 (26.5)	181 (30.1)	189 (33.7)	
Hypertension, %	405 (35.5)	420 (36.8)	376 (32.6)	384 (33.4)	353 (30.6)	0.017
CVD, %	284 (24.0)	276 (23.3)	252 (21.2)	236 (19.9)	249 (21.0)	0.093
Antihypertensive drugs, %	767 (93.5)	740 (94.9)	729 (95.7)	711 (93.8)	704 (94.8)	0.344
Hypoglycemic drugs, %	637 (68.2)	687 (71.8)	679 (70.1)	666 (67.9)	648 (67.4)	0.204
Glucose, mg/dL	157.1 (65.28)	158.9 (65.90)	155.3 (62.13)	158.3 (70.60)	159.4 (59.57)	0.827
HbA1c, %	7.36 (1.84)	7.35 (1.73)	7.40 (1.83)	7.34 (1.83)	7.31 (1.73)	0.821
Triglycerides, mg/dL	192.3 (165.5)	197.4 (218.4)	197.3 (252.1)	202.2 (175.0)	211.1 (209.3)	0.257
LDL, mg/dL	109.6 (37.2)	110.6 (37.8)	107.0 (38.7)	110.3 (37.7)	104.9 (33.8)	0.057
Serum iron, mg/dL	77.04 (31.71)	76.83 (30.59)	79.97 (32.81)	77.76 (30.75)	82.82 (34.25)	<0.001
eGFR, ml/min per 1.73 m2	80.70 (33.44)	82.35 (31.71)	84.52 (33.51)	85.62 (30.88)	88.12 (33.22)	<0.001
Energy, kcal	1077.8 (377.9)	1481.2 (388.2)	1804.8 (506.8)	2069.0 (631.7)	2534.1 (953.7)	<0.001

*PIR* poverty income ratio, *BMI* body mass index, *CVD* cardiovascular diseases, *LDL* low-density lipoprotein, *eGFR* estimated glomerular filtration rate.

Data were presented as mean (SD) or *n* (%).

**Fig. 1:**
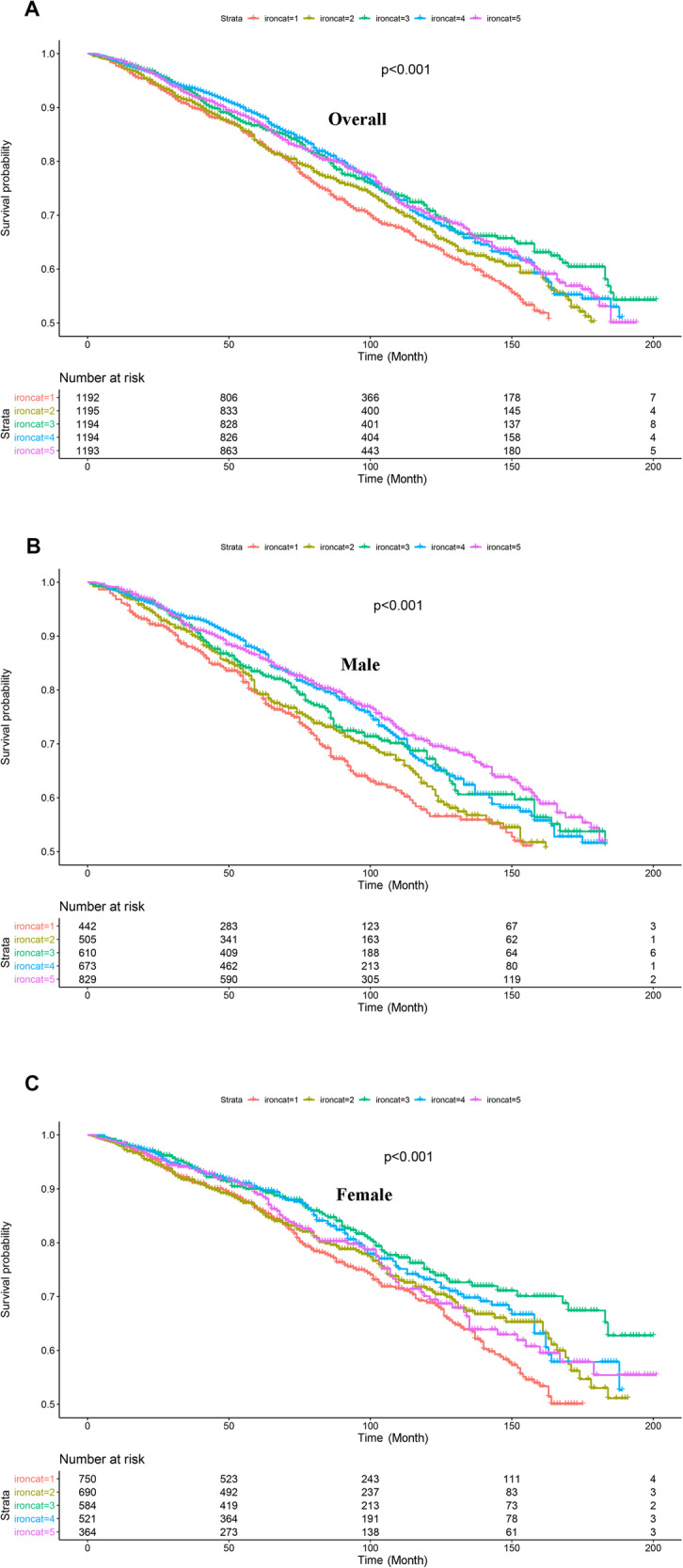
Univarite survival analysis. The Kaplan–Meier analysis of the prognostic effect of the iron intake quintile on all-cause mortality in overall populations (**A**), male population (**B**), and female population (**C**). Ironcat represented the iron intake quintile.

During 41,425 person-years of follow-up, 1497 deaths occurred. As shown in Fig. 1, Kaplan–Meier analysis suggested that low level of iron intake was associated with a higher all-cause mortality, both in males and females. After adjusting for demographics, lifestyles, and medical history, we found that iron intake between 11.1 and 14.4 was associated with the lowest all-cause mortality compared with the participants with iron intake <6.3 mg (HR 0.83, 0.70–0.99; *P* < 0.05; Table 2). The group with iron intake of 14.4–18.8 was related to a low risk of cardiovascular mortality (HR: 0.68, 0.47–1.00; *P* < 0.05), however, the relationship disappeared after multivariable adjustment. No significant association was observed between the iron intake and cancer mortality.

**Table 2:** Association of iron intake with all-cause and cause-specific mortality.

All-cause and cause-specific mortality	Cases	*N*	Model 1 HR (95% CI)	Model 2 HR (95% CI)	Model 3 HR (95% CI)
All causes					
F1	345	1192	Ref	Ref	Ref
F2	317	1195	0.87 [0.75, 1.01]	0.89 [0.76, 1.03]	0.90 [0.77, 1.06]
F3	265	1194	0.81 [0.69, 0.95]**	0.82 [0.70, 0.97]*	0.83 [0.70, 0.99]*
F4	275	1194	0.80 [0.68, 0.94]**	0.85 [0.72, 1.00]*	0.86 [0.72, 1.03]
F5	295	1193	0.80 [0.68, 0.94]**	0.86 [0.73, 1.01]	0.90 [0.73, 1.09]
Cardiovascular					
F1	73	345	Ref	Ref	Ref
F2	67	314	0.99 [0.71, 1.38]	1.00 [0.71, 1.40]	1.08 [0.76, 1.53]
F3	60	264	1.11 [0.78, 1.56]	1.14 [0.79, 1.63]	1.30 [0.88, 1.93]
F4	45	274	0.68 [0.47, 1.00]*	0.71 [0.49, 1.05]	0.83 [0.55, 1.27]
F5	61	294	0.82 [0.58, 1.16]	0.88 [0.62, 1.27]	1.09 [0.70, 1.68]
Malignant neoplasms					
F1	57	345	Ref	Ref	Ref
F2	46	314	0.89 [0.60, 1.32]	0.91 [0.61, 1.34]	0.91 [0.61, 1.37]
F3	45	264	1.03 [0.69, 1.53]	0.94 [0.62, 1.40]	0.95 [0.61, 1.47]
F4	50	274	0.98 [0.67, 1.44]	0.95 [0.64, 1.40]	0.96 [0.62, 1.48]
F5	54	294	0.96 [0.65, 1.40]	0.97 [0.66, 1.43]	0.99 [0.62, 1.59]

Model 1: adjusted for gender and age.

Model 2: adjusted for gender, age, race, education, PIR, BMI, drinking, smoking and activity.

Model 3: adjusted for gender, age, race, education, PIR, BMI, drinking, smoking, activity, hypertension, CVD, eGFR, serum iron, and energy.

*HR* hazard ratio, *CI* confidence interval.

*P < 0.05, **P < 0.01.

**Fig. 2:**
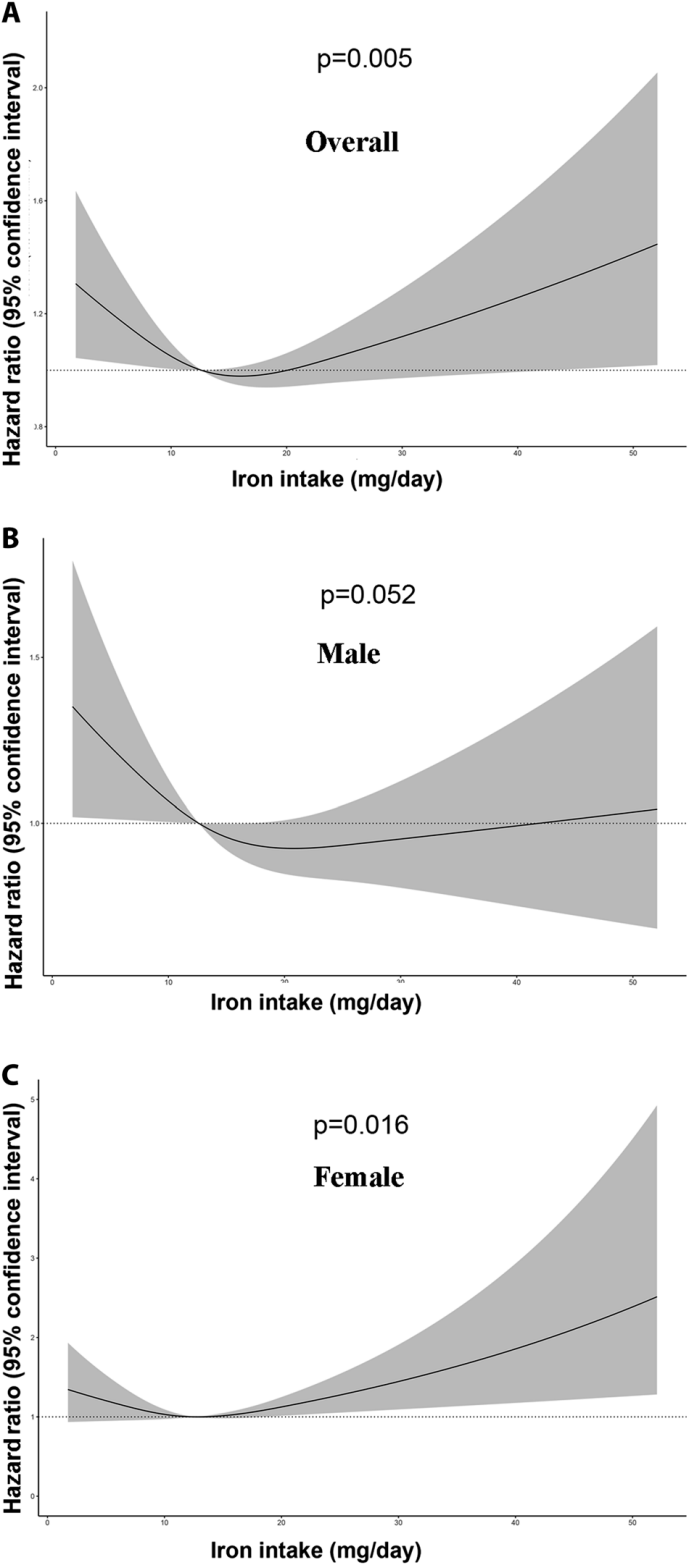
Nonlinear analyis. The restricted cubic regression between the iron intake with all-cause mortality in overall populations (**A**), male population (**B**), and female population (**C**) in fully adjusted model.

To explore the nonlinear relationship between iron intake and all-cause mortality, restricted cubic spines based on Cox regression models were performed (Fig. 2). We found a nonlinear association existed in overall (*P* = 0.005) and female (*P* = 0.016) populations. The relationship followed an L shape in men and a J shape in women. Subgroup analysis (Table 3) also showed that there was no significant interaction between baseline iron levels and iron intake (*P* = 0.296).

**Table 3:** Subgroup analysis of the association between iron intake with all-cause mortality across serum iron levels.

Serum iron levels (mg/dL)	F1	F2	F3	F4	F5	*P* for trend	*P* for interaction
<57	Ref	0.93 [0.70, 1.25]	0.88 [0.63, 1.23]	0.77 [0.55, 1.09]	0.86 [0.57, 1.28]	0.227	0.296
57–74	Ref	0.95 [0.68, 1.32]	0.65 [0.44, 0.94]*	0.98 [0.67, 1.43]	0.93 [0.62, 1.40]	0.891	
74–96	Ref	0.84 [0.60, 1.19]	1.13 [0.79, 1.62]	0.94 [0.64, 1.38]	1.04 [0.70, 1.56]	0.657	
>96	Ref	0.89 [0.64, 1.25]	0.67 [0.46, 0.95]*	0.79 [0.55, 1.14]	0.73 [0.49, 1.10]	0.116	

## Discussion

The findings of this study revealed a nonlinear association between iron intake and all-cause mortality among individuals with diabetes. Both lower and higher levels of iron intake were found to elevate the risk of all-cause mortality. Interestingly, while higher iron intake was linked to a reduced risk of all-cause mortality in men, it was associated with an increased risk in women. However, no significant correlations were observed between iron intake and mortality specifically attributable to cardiovascular disease or cancer.

Many studies have investigated the association between iron intake and the risk of mortality in the general population [13–16]. A study reported that elevated serum iron level was positively related to increased mortality risk in prediabetes [17]. What’s more, another study has shown that both low and high serum ferritin, reflecting the deficiency and redundancy of body iron stores, were associated with poor prognosis of patients with diabetes and cardiovascular disease [18]. In consistent with previous findings, our results also showed that dietary iron intake was nonlinearly associated with all-cause mortality in diabetic individuals, namely a higher or lower iron intake relating to an increased risk of all-cause mortality.

Iron plays essential roles in human physiology by contributing to the constitution of hemoglobin, facilitating DNA synthesis, and aiding in mitochondrial electron transport. Thus, maintaining moderate iron supplementation is crucial for supporting these fundamental functions in daily life [18, 19]. However, the excess iron load was detrimental to the body, causing plentiful iron deposits in the liver and heart and visceral damage [20]. The accumulation of iron has been linked to heightened oxidative stress and can disrupt insulin secretion, potentially increasing the risk of developing diabetes and cardiovascular disease (CVD). Given these associations, our findings are plausible and suggest that further research should prioritize investigating the underlying mechanisms involved in these processes. Understanding the mechanisms behind iron-related oxidative stress and its impact on insulin secretion could provide valuable insights into the prevention and management of diabetes and CVD.

Interestingly, we also noticed a gender difference in that higher iron intake decreased the mortality in males, while higher iron intake increased the mortality in females. A study also reported the gender discrepancy when exploring the relationship between dietary iron intake and the risk of diabetes. A previous study found that higher iron intake was associated with an increased risk of diabetes in men [21], which was contrary to our findings. According to a cross-sectional survey, females had fewer iron intake than males in all age [22], and reference ranges of ferritin in women were lower than that in equivalent- aged males [23]. These studies all indicated a higher iron storage in males, that was to say, males were accustomed to a relatively elevated body iron. We hypothesized that high iron intake broke the metabolic balance in females and accelerated oxidative stress, leading to increased mortality. Besides, dietary sources of iron may influence its relationship. For example, higher iron intakes reflected more red meat consumption, which were linked with diabetes and comorbidities [24]. The sex difference in the association between iron intake and mortality may be due to the fact that women have a different risk of having anemia before and after menopause [14]. However, further research was needed.

Numerous studies elaborated on the inconsistent association between iron intake and CVD and cancer mortality. A meta-analysis demonstrated that heme iron intake was positively related to coronary heart disease (CHD) incidence, and elevated serum transferrin saturation concentration was inversely relevant to CHD mortality [25], indicating the adverse role of iron in CVD mortality [26]. But some other studies clarified contrary opinions [27, 28]. It was reported that elevated iron levels increased the risk of cancer mortality [29, 30]. However, no significant statistical difference was detected in our study. Considering that many studies took serological indicators, such as ferritin, transferrin, or transferrin saturation concentration, which may directly reflect the actual body iron store while dietary iron intake may be influenced by absorption, transportation, and storage [31, 32].

The study had a relatively long follow-up time and a large sample size. Nevertheless, there were also some limitations. Firstly, other indicators, like ferritin and transferrin saturation concentration, were not adopted to evaluate the body’s iron storage. Secondly, disease history was acquired by questionnaires and interviews, which may cause recall errors. The implication of these findings is that caution should be exercised when recommending the appropriate range of iron intake for males and females with diabetes. This suggests that there may be an optimal level of iron intake for individuals with diabetes, and going above or below this threshold could potentially impact all-cause mortality. Further research and consideration are needed to establish more precise recommendations for iron intake in this specific population.

## Conclusions

In conclusion, our study has confirmed a nonlinear relationship between dietary iron intake and all-cause mortality in adults with diabetes, exhibiting a J-shaped pattern among women and an L-shaped pattern among men. We identified a threshold of 12 mg per day, indicating the need for caution when recommending appropriate ranges of iron intake for males and females, respectively. Further research is warranted to better understand the implications of these findings and to guide personalized dietary recommendations for individuals with diabetes.

## Data Availability

The original data can be obtained from NHANES (https://www.cdc.gov/nchs/nhanes/index.htm).
